# Resveratrol Induces the Expression of Interleukin-10 and Brain-Derived Neurotrophic Factor in BV2 Microglia under Hypoxia

**DOI:** 10.3390/ijms150915512

**Published:** 2014-09-02

**Authors:** Juhyun Song, So Yeong Cheon, Wonsug Jung, Won Taek Lee, Jong Eun Lee

**Affiliations:** 1 Department of Anatomy, Yonsei University College of Medicine, Seoul 120-752, Korea; E-Mails: sjh1008@yuhs.ac (J.S.); rigel110@hanmail.net (S.Y.C.); inskull@yuhs.ac (W.T.L.); 2 BK21 Plus Project for Medical Sciences, and Brain Research Institute, Yonsei University College of Medicine, Seoul 120-752, Korea; 3 Department of Anatomy, Gachon University School of Medicine, Incheon 406-799, Korea; E-Mail: wonsug@gmail.com

**Keywords:** resveratrol, microglia, hypoxia, nuclear factor kappa-light-chain enhancer of activated B cells (NF-κB), interleukin-10 (IL-10), brain-derived neurotrophic factor (BDNF)

## Abstract

Microglia are the resident macrophages of the central nervous system (CNS) and play an important role in neuronal recovery by scavenging damaged neurons. However, overactivation of microglia leads to neuronal death that is associated with CNS disorders. Therefore, regulation of microglial activation has been suggested to be an important target for treatment of CNS diseases. In the present study, we investigated the beneficial effect of resveratrol, a natural phenol with antioxidant effects, in the microglial cell line, BV2, in a model of hypoxia injury. Resveratrol suppressed the mRNA expression of the pro-inflammatory molecule, tumor necrosis factor-α, and promoted the mRNA expression of the anti-inflammatory molecule, interleukin-10, in BV2 microglia under hypoxic conditions. In addition, resveratrol inhibited the activation of the transcription factor, nuclear factor kappa-light-chain enhancer of activated B cells (NF-κB), which is upstream in the control of inflammatory reactions in hypoxia-injured BV2 microglia. Moreover, resveratrol promoted the expression of brain-derived neurotrophic factor (BDNF) in BV2 microglia under hypoxic stress. Overall, resveratrol may promote the beneficial function of microglia in ischemic brain injury.

## 1. Introduction

Microglia, the resident macrophages in the central nervous system (CNS), are activated in CNS injury and contribute to the regulation of immune responses [1,2]. Upon activation, microglia produce various molecules that are associated with CNS diseases [3,4] and also participate in the regulation of CNS homoeostasis by phagocytizing apoptotic neurons [3,5]. The beneficial function of microglia is considered to be associated with the phagocytosis that is involved in neuronal survival [6,7,8], whereas the detrimental function of microglia is from the secretion of pro-inflammatory cytokines [9]. Many studies have investigated resveratrol’s effect on the beneficial function of microglia in injured tissue. Resveratrol (3,5,4'-trihydroxy-trans-stilbene), a natural non-flavonoid, poly-phenolic compound found in the skin of grapes and red wine [10], is commonly recognized for its anti-aging [11], anti-inflammatory, antioxidant, and anti-cancer effects [12]. In the CNS, resveratrol increases the antioxidant capacity of neuronal cells and reduces lipid peroxidation in ischemia [13,14,15,16,17]. Resveratrol exerts anti-inflammatory effects in microglia by suppressing the expression of a variety of pro-inflammatory cytokines and signaling molecules [18]. In addition, resveratrol exerts protective effects by modulating the activity of the transcription factor, nuclear factor kappa-light-chain enhancer of activated B cells (NF-κB) [15,19]. NF-κB is an important transcription factor for the induction of inflammatory mediators [20]. Several studies have demonstrated that NF-κB is a major regulatory factor in the pathogenesis of microglia-mediated neuroinflammation [21,22]. Many studies have suggested that gene expression of anti-inflammatory cytokines such as interleukin (IL)-10 [23,24] and pro-inflammatory cytokines such as tumor necrosis factor (TNF)-α, IL-1β, and monocyte chemotactic protein-1 (MCP-1) is controlled by the activation of NF-κB [25,26,27,28,29,30,31]. Among the anti-inflammatory cytokines, IL-10 has a variety of beneficial effects in the CNS. IL-10 is synthesized in microglia and astrocytes [32] and inhibits the microglial secretion of pro-inflammatory cytokines such as TNF-α [33,34]. IL-10 delays the onset and reduces the severity of experimental autoimmune encephalomyelitis (EAE) in mice [35], is involved in the clinical pathology of Alzheimer’s disease [36], and reduces the infarct size in the brain of an ischemic mouse model [37,38]. IL-10 inhibits apoptotic pathways by blocking NF-κB binding to DNA [39]. The regulation of IL-10 secretion by microglia is linked to the activation of NF-κB signaling and may be increased by resveratrol. Moreover, one of the beneficial functions of microglia is the secretion of neurotrophic factors, which contribute to the survival of neurons, astrocytes, and oligodendrocytes [40]. The absence of neurotrophic factors results in neuronal death and neurodegenerative diseases [41,42]. Among the neurotrophic factors, brain-derived neurotrophic factor (BDNF) is secreted in the hippocampus, cerebral cortex, and basal forebrain [43]. BDNF has beneficial effects on cognition, learning, and memory formation by modulating synaptic plasticity [44,45,46,47]. Microglia may regulate learning-dependent synapse formation by modulating BDNF secretion [48,49]. Several studies have demonstrated that microglial BDNF is present in various regions of the CNS during the course of several neurological disorders such as traumatic injury [50,51] and ischemia [49]. Several studies have reported that resveratrol increases BDNF gene expression in the hippocampus of rat brain [52,53,54]. Here, we examined the effect of resveratrol on microglia under hypoxic conditions. We investigated whether resveratrol contributes to an increase of IL-10, BDNF expression and activation of NF-κB. These findings may indicate that resveratrol plays a neuroprotective role in hypoxic brain injury.

## 2. Results and Discussion

### 2.1. Resveratrol Increases Cell Viability of Microglia under Hypoxic Conditions

**Figure 1:**
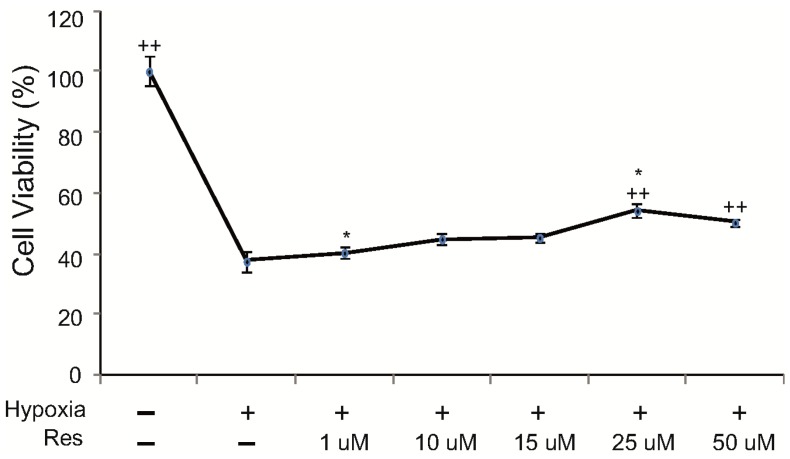
The measurement of cell viability (MTT assay). To check the cell viability in BV2 microglia, we conducted a MTT assay. This data showed that BV2 microglia in the hypoxia injury group exhibited a decreaseof cell viability compared to the normal control group. BV2 microglia in all resveratrol pre-treatment groups exhibited increase of cell viability after hypoxia injury compared to the hypoxia injury group without resveratrol. Among the resveratrol concentrations tested, 25μM resveratrol showed >50% cell viability. Resveratrol (25μM) increased the cell viability of hypoxia-injured microglia. Data were expressed as mean ± S.E.M, and were analyzed statistically using one-way Analysis of variance (ANOVA), followed by Bonferroni’s *post hoc* and *t*-test. ^*^p < 0.05 (the *t*-test compared tothe hypoxia group), **^++ ^***p* < 0.01 (ANOVA test). **Res**: resveratrol pre-treatment for 3 h, **Hypoxia**: hypoxia injury for 4 h.

To investigate the cell viability of BV2 microglia under hypoxic injury, we conducted the 3-(4,5-dimethylthiazol-2-yl)-2,5-diphenyl tetrasodium bromide (MTT) assay. The cell viability of hypoxia injured BV2 microglia was increased by resveratrol pre-treatment (1–50 μM). After pre-treatment with 25 μM resveratrol, the cell viability of hypoxia-injured BV2 microglia was over 50%. Because pre-treatment with 25 μM resveratrol showed the highest cell viability among all concentrations of resveratrol (Figure 1), we used 25 μM resveratrol in all the following experiments.

Several studies have demonstrated that resveratrol increases cell viability of microglia with lipopolysaccharide-induced oxidative stress [55,56]. However, research on the effect of resveratrol on microglia under hypoxic injury was not reported until now. Our results show that cell viability of hypoxia-injured BV2 microglia is increased by resveratrol treatment.

### 2.2. Microglia Activation Is Attenuated by Resveratrol under Hypoxia Injury

**Figure 2:**
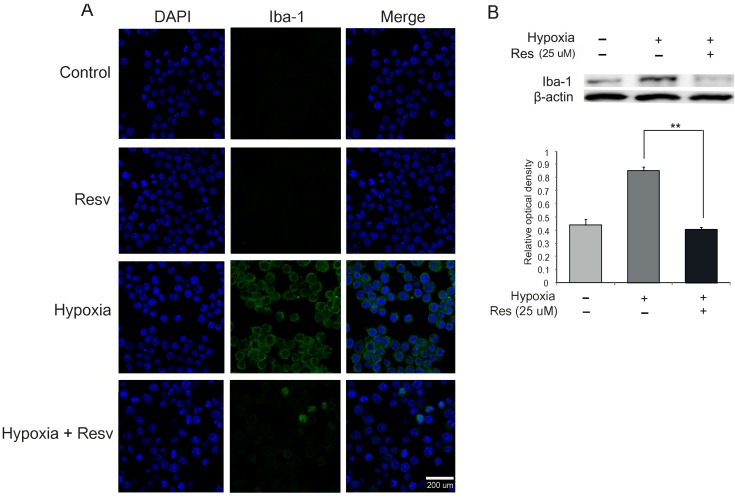
The expression of Iba-1in hypoxia-injured BV2 microglia. (**A**) The level of Iba-1 (a marker of activated microglia) was evaluated by immunocytochemistry. This image shows that the expression of Iba-1 in the hypoxia group exposed to hypoxia was increased compared to the control group (no hypoxia). The resveratrol (25 μM) pre-treatment group (Hypoxia + Resv) showed a decreaseof Iba-1 expression compared with the hypoxia group. Resveratrol (25 μM) decreased the activation of microglia under hypoxic conditions; (**B**) Western blotting data showed that the protein level of Iba-1 was increased in the hypoxia group compared to the control group. Resveratrol (25 μM) decreased the protein level of Iba-1 in BV2 microglia under hypoxic conditions. β-actin was used as an internal, loading control (mean ± S.E.M., *n* = 3).^**^p < 0.01 (compared to the hypoxia group). **Control**: normal control group, **Hypoxia**: hypoxia injury group for 4 h, **Resv**: resveratrol (25 μM) treatment group under normal condition, **Hypoxia + Resv**: resveratrol (25 μM) pre-treatment before 4 h hypoxia injury, **Res** (**25 μM**): 25 μM resveratrol pre-treatment for 3 h. 4',6-diamidino-2-phenylindole (DAPI): blue, Ionized calcium binding adaptor molecule 1 (Iba-1): green, Scale bar: 200 µm.

To examine the activation of microglia, we conducted immunochemical analysis using a marker of activated microglia (ionized calcium binding adaptor molecule 1 (Iba-1)) in all groups. The expression of Iba-1 was increased in the hypoxia group compared to the control group (no hypoxia) (Figure 2A). Resveratrol (25 μM) reduced the activation of microglia compared to the hypoxia group in spite of hypoxic injury (Figure 2A). In addition, to confirm the protein level of Iba-1, we performed western blotting in all groups. The protein level of Iba-1 was greatly increased in the hypoxia injury group compared with the control group (Figure 2B). In hypoxic stress, resveratrol (25 μM) attenuated the protein levels of Iba-1 in BV2 microglia compared to the hypoxia group (Figure 2B).

Iba-1 protein is expressed in microglia [57], and is a microglia-specific calcium-binding protein that also participates in phagocytosis [58]. Ito *et al.* [59] demonstrated that Iba-1 expression is related to microglial activation in the ischemic brain, and the physiological roles of activated microglia can be evaluated by determining the expression of Iba-1 [59,60,61]. Based on our results, we concluded that hypoxia led to the activation of microglia and that resveratrol (25 μM) reduced the activation of hypoxia-injured microglia. Thus, resveratrol may attenuate the activation of microglia in hypoxic brain injury.

### 2.3. Resveratrol Modulates mRNA Expression of IL (Interleukin)-10 and TNF (Tumor Necrosis Factor)-α in Hypoxia-Injured Microglia

**Figure 3:**
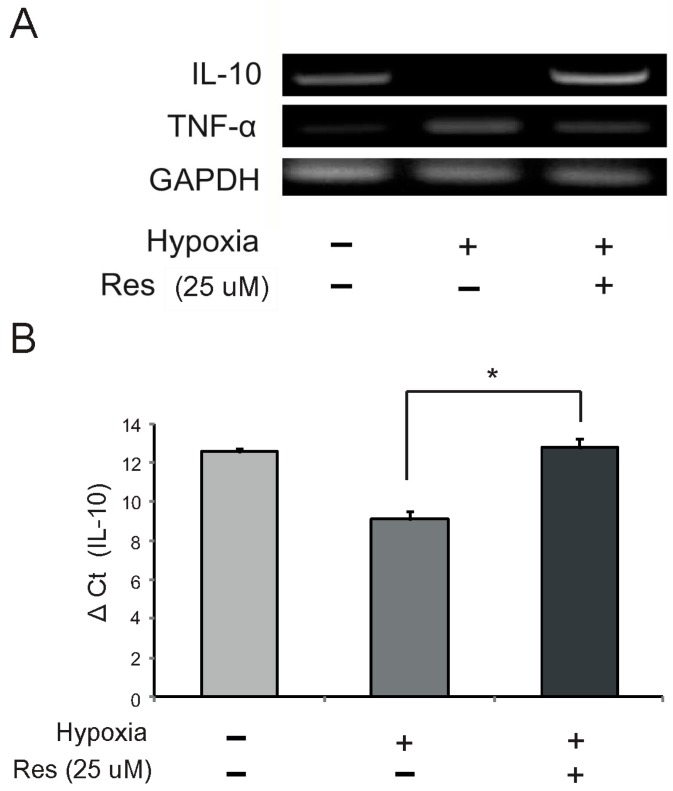
The mRNA levels of IL (interleukin)-10 and TNF (tumor necrosis factor)-α in hypoxia-injured BV2 microglia. The mRNA levels of IL-10 and TNF-α were measured using reverse transcription PCR (**A**) and quantitative real time PCR (**B**). (**A**) The mRNA level of IL-10 was decreased in the hypoxia injury group compared to the normal control group. Resveratrol (25 μM) increased the mRNA level of IL-10 in hypoxia-injured microglia. The mRNA level of TNF-α was increased in the hypoxia injury group compared to the normal control group. Resveratrol (25 μM) reduced mRNA level of TNF-α in hypoxia-injured microglia; (**B**) Δ*C*_t_ value also shows that resveratrol (25 μM) increased mRNA levels of IL-10 under hypoxia injury. GAPDH was used as a control (mean ± S.E.M., *n* = 3). ^*^p < 0.05 (compared to the hypoxia group).

To examine the mRNA levels of IL-10 and TNF-α in microglia following resveratrol pretreatment under hypoxia injury, we conducted reverse transcription—PCR and quantitative real time—PCR. The mRNA level of IL-10 was reduced in the hypoxia injury group (Figure 3A,B), whereas the mRNA level of TNF-α was increased in this group (Figure 3A). Resveratrol (25 μM) increased the mRNA level of IL-10 (Figure 3A,B) and attenuated the mRNA level of TNF-α in hypoxic conditions (Figure3A). Δ*C*_T_ value of IL-10 shows that the amount of IL-10 mRNA was increased in the hypoxia with resveratrol pre-treatment group compared to the hypoxic injury group (Figure 3B).

Resveratrol has been reported to inhibit TNF-α secretion from cells [62,63,64]. In the CNS, IL-10 may exert neuroprotective effects and stimulate recovery of neurite outgrowth and down-regulation of microglial nitric oxide production [65,66,67]. IL-10 is regarded as a potential therapeutic target because of its anti-inflammatory and immunosuppressive effects [39,68,69,70]. After CNS injuries, IL-10 reduces the number of apoptotic cells [39] and contributes to the enhancement of learning and memory [71]. In addition, several studies demonstrated that IL-10 inhibits the secretion of TNF-α in microglia [33,34]. Considering these proven beneficial effects of IL-10 in the CNS, our results suggest that resveratrol may play a beneficial role by promoting IL-10 expression and suppressing TNF-α expression in hypoxic brain injury.

### 2.4. Resveratrol Suppression of NF-κB (Nuclear Factor Kappa-Light-Chain Enhancer of Activated B Cells) Activation in Hypoxia Injured Microglia

**Figure 4:**
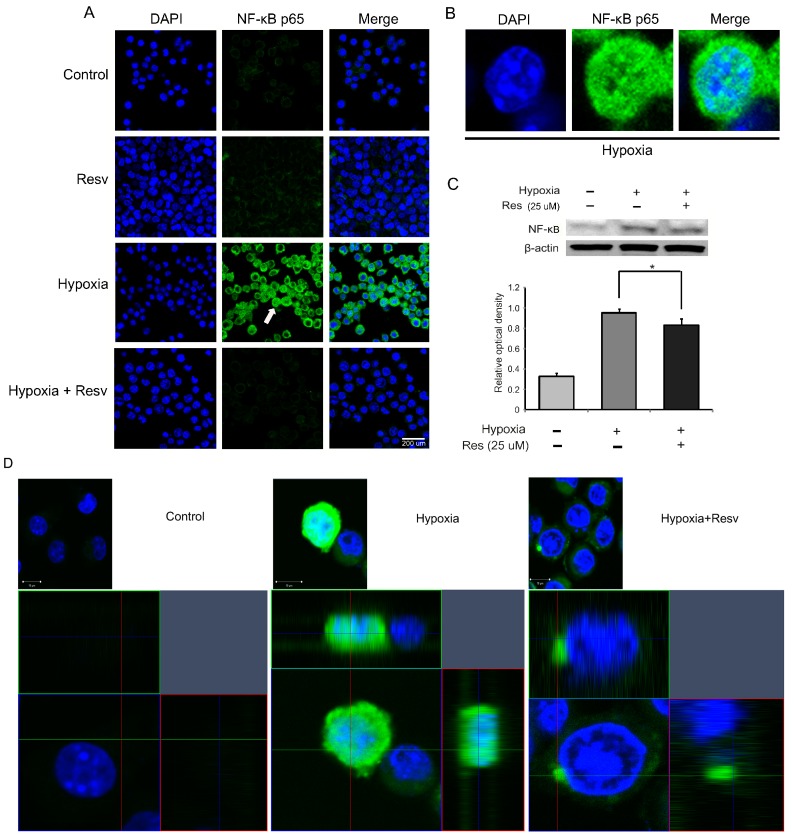
Measurement of NF-κB activation in resveratrol pre-treated BV2 microglia under hypoxic conditions. (**A**) The activation of NF-κB was evaluated with immunocytochemistry. This image showed that the translocation of NF-κB p65 to the nucleus of BV2 microglia in the hypoxia group was increased compared with the control group. The resveratrol (25 μM) pre-treatment group (Hypoxia + Resv group) showed suppression of NF-κB p65 translocation to the nucleus compared with the hypoxia group. Resveratrol (25 μM) decreased the activation of NF-κB under hypoxic conditions; (**B**) The high magnification image at the white arrow site in the hypoxia group shows that NF-κB p65 enters into the nucleus; (**C**) Western blotting data showed that the protein level of NF-κBp65 was increased in the hypoxia group compared to the control group. The protein level of NF-κBp65 was decreased in the hypoxia + resv group compared to the hypoxia group. β-actin was used as an internal, loading control (mean ± S.E.M., *n* = 3). ^*^p < 0.05 (compared to the hypoxia group);and (**D**) The hypoxia group shows the translocation of NF-κBp65 into the nucleus compared with the normal group owing to the hypoxia injury. Resveratrol inhibited the translocation of NF-κBp65 into nucleus in spite of hypoxia injury. **Control**: normal control group, **Hypoxia**: hypoxia injury group for 4 h, **Resv**: resveratrol (25 μM) treatment group under normal condition, **Hypoxia + Resv**: resveratrol (25 μM) pre-treatment before 4 h hypoxia injury, **Res** (**25 μM**): 25 μM resveratrol pre-treatment for 3 h. 4',6-diamidino-2-phenylindole (DAPI): blue, Nuclear factor-kappa B (NF-κB): green, Scale bar: 200 µm.

To investigate the activation of NF-κB in hypoxia injured microglia, we determined the translocation of NF-κB to the nucleus in BV2 microglia using immunochemical analysis. The expression of NF-κB p65 was increased in the hypoxia group compared to the control group (Figure 4A). Also, in the hypoxia group, NF-κB in the cytosol was translocated to the nucleus of microglia by hypoxic injury (Figure 4A,B). Resveratrol (25 μM) inhibited the activation of NF-κB in hypoxia-injured microglia (Figure 4A,B). In addition, we examined the protein level of NF-κB in hypoxia-injured BV2 microglia using western blot analysis (Figure 4C). The protein level of NF-κB, which was increased by hypoxia injury, was significantly inhibited by pre-treatment with resveratrol (Figure 4C).

Activation of NF-κB plays a central role in inflammation and exerts multiple effects in various cellular responses [72]. Several studies have demonstrated that resveratrol regulates the activity of apoptotic transcription factors and regulates inflammatory responses by modulating NF-κB [73,74,75]. Taken together, our data indicate that resveratrol may inhibit neuronal inflammatory responses by suppressing the activation of NF-κB in microglia under ischemic brain injury. In addition, considering Figure 3 data in this study, following resveratrol pre-treatment, NF-κB may contribute to an increase of IL-10 expression and a decrease of TNF-α expression in hypoxia-treated microglia.

### 2.5. The Expression of BDNF (Brain-Derived Neurotrophic Factor) in Resveratrol Pre-Treated Microglia under Hypoxia Stress

**Figure 5:**
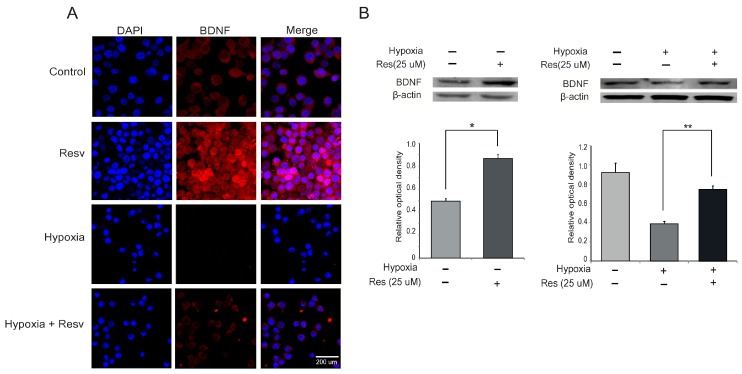
The measurement of BDNF (brain-derived neurotrophic factor) expression by resveratrol in hypoxia injured microglia. (**A**) The expression of BDNF (a neurotrophic factor) was evaluated by immunocytochemistry. This image showed that the expression of BDNF in the hypoxia group exposed to hypoxia injury was attenuated compared to the control group. The resveratrol (25 μM) treatment group (Resv group) shows that resveratrol (25 μM) increased the expression of BDNF under normal conditions. Also, the resveratrol (25 μM) pre-treatment group (Hypoxia + Resv group) showed increases of BDNF expression compared with the hypoxia group; (**B**) Western blotting data showed that the protein level of BDNF was decreased in the hypoxia group compared to the control group. The protein level of BDNF was increased in the resveratrol (25 μM) treatment group compared to the control group. The protein level of BDNF was increased in the hypoxia + resv group compared to the hypoxia group. β-actin was used as an internal, loading control (mean ± S.E.M., *n* = 3). ^*^p < 0.05, ^**^p < 0.01 (compared to the hypoxia group). **Control**: normal control group, **Hypoxia**: hypoxia injury group for 4 h, **Resv**: resveratrol (25 μM) treatment group under normal condition, **Hypoxia + Resv**: resveratrol (25 μM) pre-treatment before 4 h hypoxia injury, **Res** (**25 μM**): 25 μM resveratrol pre-treatment for 3 h. 4',6-diamidino-2-phenylindole (DAPI): blue, Brain-derived neurotrophic factor (BDNF): red, Scale bar: 200 μm.

**Figure 6:**
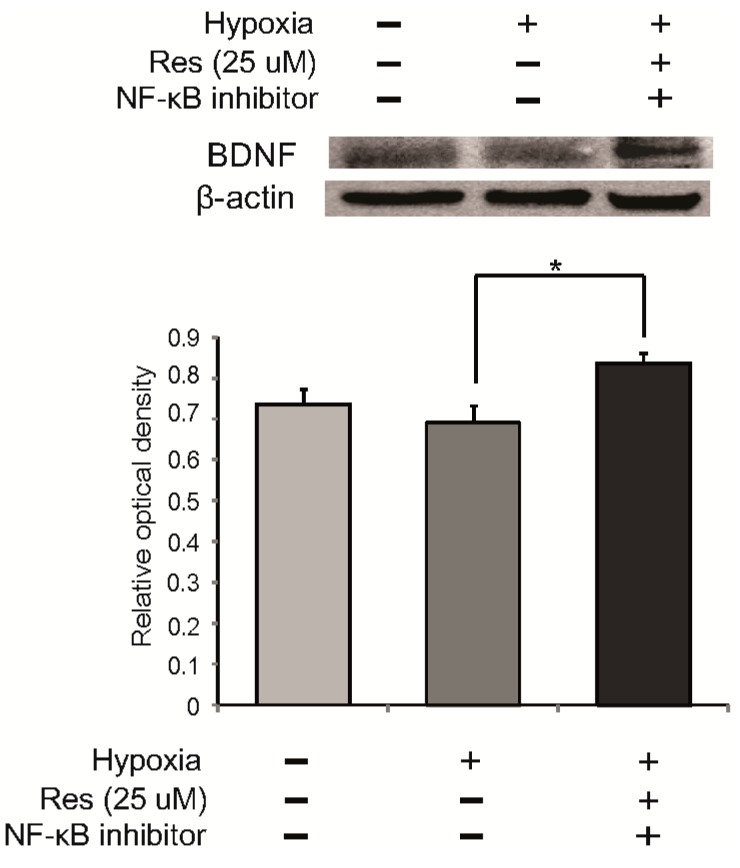
The measurement of BDNF expression in resveratrol pre-treated microglia with NF-κB inhibitor. To confirm the relationship between BDNF expression and NF-κB activation in resveratrol pre-treatment, we examined the protein level of BDNF in BV2 microglia using western blot analysis after 100nM NF-κB inhibitor (Santa Cruz, CA, USA) pre-treatment at 3 h before hypoxia injury. After hypoxia injury, the protein level of BDNF was increased in the 25 μM resveratrol with 100 nM NF-κB inhibitor pre-treatment group. β-actin was used as an internal, loading control (mean ± S.E.M., *n* = 3). ^*^p < 0.05 (compared to the hypoxia group).

To examine secretion of a neurotrophic factor following resveratrol pre-treatment in hypoxia injured microglia, we examined the expression of BDNF using immunochemical analysis. In the hypoxia group, the expression of BDNF was attenuated compared to the control group, whereas the expression of BDNF was enhanced by resveratrol (25 μM) despite the hypoxia stress (Figure 5A). In addition, we measured the protein level of BDNF using western blot analysis in all groups (Figure 5B). BDNF expression was attenuated in hypoxia-injured microglia compared to the control group, whereas BDNF expression was increased by resveratrol in microglia in spite of hypoxia stress (Figure 5B). To confirm whether resveratrol is related to BDNF expression through NF-κB signaling, we pretreated the NF-κB inhibitor in microglia at 3 h before hypoxia stress (Figure 6). The expression of BDNF by resveratrol in hypoxia-injured microglia was increased by NF-κB inhibitor pre-treatment (Figure 6). This result indicates that resveratrol may be involved in the expression of BDNF through NF-κB signaling.

BDNF plays an important role in the survival of neuronal cells [76] and also enhances cognition, learning, and memory ability [44]. Several studies have demonstrated that resveratrol improves BDNF expression in hippocampal brain regions [54,77] and in astroglia [78]. Taken together, our data show that resveratrol promotes the expression of BDNF in hypoxia-injured microglia. Also, the enhancement of BDNF expression by resveratrol may be associated with NF-κB signaling. Thus, resveratrol may exert the beneficial effect by modulating the expression of BDNF in hypoxic brain injury. Our study has some limitations. We investigated BDNF expression only in BV2 microglia, not in hippocampal brain regions, and we did not examine memory function, neuronal survival, or synapse formation in the presence of BDNF. However, our results indicate that resveratrol may be beneficial in hypoxic brain injury by enhancing BDNF expression in microglia.

## 3. Experimental Section

### 3.1. Cell Culture and Drug Treatment

Murine BV2 microglial cells were obtained from Prof. Eun-hye Joe (Ajou University School of Medicine, Chronic Inflammatory Disease Research Center) and cultured in Dulbecco Modified Eagle Medium (DMEM) (Gibco, Grand Island, NY, USA) supplemented with 10% fetal bovine serum (Gibco) and 100 μg/mL penicillin-streptomycin (Gibco) at 37 °C in humidified atmosphere containing 5% CO_2_. Resveratrol was purchased from Sigma Aldrich (Sigma, St. Louis, MO, USA). BV2 microglial cells were pretreated with resveratrol at 3 h before hypoxia stress. BV2 microglial cells were pretreated with NF-κB inhibitor (Santa Cruz biotechnology, Santa Cruz, CA, USA) that inhibits translocation of the NF-κB active complex into the nucleus at 3 h before hypoxia stress.

### 3.2. Hypoxic Conditions

Murine BV2 microglial cells were transferred to an anaerobic chamber (Forma Scientific, Marietta, OH, USA) (O_2_ tension, 0.1%) and washed 3 times with phosphate-buffered saline (PBS). Then, culture medium was replaced with de-oxygenated, glucose-free balanced salt solution, and cells with or without resveratrol pre-treatment were incubated for 4 h [79].

### 3.3. Cell Viability Assay (MTT Assay)

BV2 microglial cells (2 × 10^5^ cells/mL) were seeded in 96-well plates to monitor all experimental conditions, including resveratrol pre-treatment (3 h) and oxygen-glucose deprivation injury (4 h). Next, cells were rinsed twice with PBS, and culture medium was replaced with serum-free medium. Then, 100 μL of 3-(4,5-dimethylthiazol-2-yl)-2,5-diphenyltetrazolium bromide (MTT) (Sigma) solution (5 mg/mL in PBS) was added per well. After 1 h of incubation, medium was removed, and dimethyl sulfoxide was added to solubilize the purple formazan product of the MTT reaction. The supernatant from each well was analyzed using an enzyme-linked immunosorbent array (ELISA) plate reader at a wavelength of 570 nm, with background subtraction at 650 nm. All experiments were repeated at least three times. Cell viability in control medium without any treatment was considered 100%. Cell viability was reported as the value relative to the control group.

### 3.4. Western Blot Analyses

Protein (50 µg) was extracted from BV2 microglia cultures, and equal amounts were electrophoresed on 10%–12% SDS-polyacrylamide gels. Separated proteins were electrotransferred to Immunobilon-NC membranes (Millipore, Massachusetts, MA, USA), which were blocked for 1 h at room temperature with 5% skim milk in Tris-buffered saline and 0.1% Tween-20 (TBST). The primary antibodies used were NF-κB p65 (1:2000, Millipore), BDNF (1:2000, Millipore), Iba-1 (1:2000, Santa Cruz), and β-actin (1:1000, Santa Cruz). Blots were incubated with the primary antibodies overnight at 4 °C. Membranes were washed three times (5 min each) with TBST. The secondary antibodies were anti-rabbit and anti-mouse (1:2000, New England Biolabs, Ipswich, MA, USA) and were incubated for 1 h at room temperature. After washing with TBST (0.05% Tween 20) three times, immunoreactive signals were detected using chemiluminescence and an ECL detection system (Amersham Life Science, Buckinghamshire, UK) with the LAS 4000 program.

### 3.5. Reverse Transcription—PCR

To examine the expression of IL-10 and TNF-α in resveratrol pre-treated BV2 cells under hypoxic condition, RT-PCR was performed using IL-10 and TNF-α primers. Briefly, samples were lysed with Trizol reagent (Invitrogen, Carlsbad, CA, USA), and total RNA was extracted according to the manufacturer’s protocol. cDNA synthesis from mRNA and sample normalization were performed. PCR was performed using the following thermal cycling conditions: 10 min at 95 °C; 40 cycles of denaturing at 95 °C for 15 s, annealing for 30 s at 56 °C (IL-10) or 65 °C (TNF-α), elongation at 72 °C for 30 s; final extension for 10 min at 72 °C, and held at 4 °C. PCR was performed using the following primers (5' to 3'); IL-10 forward (F): CCAAGCCTTATCGGAAATGA, reverse (R): TTTTCACAGGGGAGAAATCG, TNF-α—F: CAAGGGACAAGGCTGCCCCG, R: GCAGGGGCTCTTGACGGCAG, GAPDH—F: GGCATGGACTGTGGTCATGAG, R: TGCACCACCAACTGCTTAGC. PCR products were electrophoresed in 1.5% agarose gels and stained with ethidium bromide.

### 3.6. Quantitative Real Time—PCR

To examine the amount of IL-10 mRNA in resveratrol pre-treated BV2 cells under hypoxic condition, quantitative real time PCR was performed using the IL-10 primer. Total cellular RNA was extracted from the BV2 microglia cells using Trizol reagent (Invitrogen) according to the manufacturer’s instructions. BV2 microglia. Poly(A) was added using poly(A) polymerase (Ambion, Foster City, CA, USA). One Step SYBR^®^ Prime Script TM RT-PCR Kit II (Takara, Shiga, Japan) was used to conduct qRT-PCR. PCR was performed using the following primers (5' to 3'); IL-10 forward (F): CCAAGCCTTATCGGAAATGA, reverse (R): TTTTCACAGGGGAGAAATCG, GAPDH—F: GGCATGGACTGTGGTCATGAG, R: TGCACCACCAACTGCTTAGC. Denaturing was carried out at 95 °C for 3 min; 40 cycles of 95 °C for 20 s; annealing at 60 °C for 20 s and extension at 72 °C for 20 s. At each extension step at 72 °C, fluorescence was detected at 585 nm. The expression of IL-10 was assessed using an ABI prism 7000 Real-Time PCR System (Life Technologies Corporation, Carlsbad, CA, USA) and analyzed with comparative *C*_t_ quantification [80]. GAPDH was amplified as an internal control. The *C*_t_ values of GAPDH were subtracted from the *C*_t_ values of the IL-10 genes (Δ*C*_t_). The Δ*C*_t_ values of the treated cells were compared with the Δ*C*_t_ values of the untreated cells.

### 3.7. Immunocytochemistry

The expression of NF-κB p65, Iba-1 and BDNF in BV2 cells was examined by immunocytochemistry. Cells in all experimental groups were washed three times with PBS, fixed with 4% paraformaldehyde for 3 h, and then washed with PBS. BV2 cells were permeabilized with 0.025% Triton X-100 and blocked for 1 h at room temperature with dilution buffer (Invitrogen). Primary antibodies, anti-rabbit NF-κB p65 (1:500, Millipore), anti-rabbit Iba-1 (1:500, Santa Cruz), and anti-rabbit BDNF (1:500, Millipore), were prepared in dilution buffer, added to samples, and incubated for 3 h at room temperature. Primary antibody was then removed, and cells were washed three times for 3 min each with PBS. Next, samples were incubated with Flurescein isothiocyanate (FITC)-conjugated goat anti-rabbit (1:200, Jackson Immunoresearch, West Grove, PA, USA), Rhodamine-conjugated goat anti-rabbit (1:200, Jackson Immunoresearch) for 2 h at room temperature. Cells were washed again three times for 3 min each with PBS and stained with 1 µg/mL DAPI (1:100, Invitrogen) for 10 min at room temperature. Fixed samples were imaged using a Zeiss LSM 700 confocal microscope (Carl Zeiss, Thornwood, NY, USA).

### 3.8. Statistical Analyses

Statistical analyses were carried out using SPSS 18.0 software (IBM Portsmouth, IBM North Harbour, Portsmouth, Hampshire, UK). All data are expressed as mean ± S.E.M. Statistical significance in intergroup differences was determined by one-way analysis of variance, followed by Bonferroni *post hoc* multiple comparison test. Statistical significance with the hypoxia only group was determined by *t*-test. Each experiment included at least three replicates per condition. Differences were considered significant at ^*^p < 0.05, ^**^p < 0.01 (the *t*-test compared to the hypoxia group), **^++ ^***p* < 0.01 (ANOVA test).

## 4. Conclusions

Overall, this study shows that resveratrol promoted the expression of BDNF and IL-10 and inhibited the expression of TNF-α in hypoxia-injured BV2 microglia. In addition, NF-κB signaling may participate in the expression of BDNF in hypoxia-injured microglia. The findings of this study may contribute to a better understanding of the mechanisms by which resveratrol modulates the beneficial function of microglia during hypoxic injury. Hence, resveratrol may act as a beneficial regulator of microglia in hypoxic brain injury.
